# Prime Editing-Based Functional Characterization Supports a Likely Pathogenic Interpretation of *NF1* c.6394T>C (p.Ser2132Pro)

**DOI:** 10.3390/genes17070838

**Published:** 2026-07-21

**Authors:** Jiayu Wu, Guangyu Li, Song Liu, Chenyu Ma, Xiaoyue Wang

**Affiliations:** 1 State Key Laboratory of Complex, Severe, and Rare Diseases, Center for Bioinformatics, National Infrastructures for Translational Medicine, Institute of Clinical Medicine, Peking Union Medical College Hospital, Chinese Academy of Medical Sciences & Peking Union Medical College, Beijing 100730, China; 2 State Key Lab of Molecular Oncology, National Cancer Center/National Clinical Research Center for Cancer/Cancer Hospital, Chinese Academy of Medical Sciences & Peking Union Medical College, Beijing 100021, China; 3 Center for Bioinformatics, National Infrastructures for Translational Medicine, Institute of Clinical Medicine, Peking Union Medical College Hospital, Chinese Academy of Medical Sciences & Peking Union Medical College, Beijing 100730, China

**Keywords:** neurofibromatosis type 1, *NF1*, RAS signaling, variant reclassification, prime editing

## Abstract

**Background/Objectives**: *NF1* encodes neurofibromin, a RAS-GTPase-activating protein (GAP), and heterozygous loss-of-function variants cause neurofibromatosis type 1. Missense variants outside the GAP-related domain (GRD) are difficult to classify because supporting functional evidence is limited. *NF1* c.6394T>C (p.Ser2132Pro) is currently listed in ClinVar as a variant of uncertain significance. We examined its functional consequences and evaluated whether the resulting evidence supports a likely pathogenic interpretation under the ACMG/AMP framework. **Methods**: We evaluated p.Ser2132Pro using population databases, evolutionary conservation, calibrated in silico predictors, and structural mapping onto the full-length cryo-EM model of neurofibromin. The variant was then introduced at the endogenous *NF1* locus in HEK293T and A375 cells by prime editing, and we measured neurofibromin abundance, transcript levels, RAS-GTP dynamics, and MAPK pathway reactivation after PLX4032 treatment. Evidence was integrated under the ACMG/AMP framework. **Results**: p.Ser2132Pro was absent from population databases, affected a highly conserved residue buried within the C-terminal HEAT domain, and received concordant deleterious predictions from calibrated in silico tools. At the endogenous locus, p.Ser2132Pro reduced neurofibromin abundance by 88–95% in both cell models while *NF1* transcript levels were only modestly reduced, impaired RAS-GTP signal termination, doubled steady-state RAS-GTP in A375 cells, and produced 5-fold higher ERK phosphorylation than the non-targeting control under PLX4032 treatment. **Conclusions**: These findings support a cellular loss-of-function effect of p.Ser2132Pro characterized primarily by reduced neurofibromin abundance with impaired neurofibromin-dependent RAS regulation. Under a conservative ACMG/AMP interpretation, the combined evidence supports a Likely Pathogenic interpretation of *NF1* c.6394T>C (p.Ser2132Pro).

## 1. Introduction

Next-generation sequencing identifies millions of human genetic variants, but variant interpretation has not kept pace [1]. A large number of missense variants in ClinVar remain classified as variants of uncertain significance (VUS) [2,3], which can preclude molecular diagnosis, complicate genetic counseling, and limit access to targeted therapy [4].

This challenge is particularly pronounced for *NF1*, a clinically important human disease gene. *NF1* encodes neurofibromin, a GTPase-activating protein (GAP) that accelerates GTP hydrolysis on RAS and thereby suppresses downstream MAPK and PI3K–mTOR pathways [5]. Germline pathogenic *NF1* variants cause neurofibromatosis type 1 (NF1), one of the most common autosomal dominant tumor-predisposition syndromes, with an incidence of approximately 1 in 2500–3000 [6]. Molecular diagnosis of NF1 has been incorporated into clinical practice guidelines for the disease [7]. Somatic *NF1* mutations occur across a broad range of malignancies, including melanoma, glioblastoma, ovarian cancer, and lung cancer [8]. Beyond its role in primary tumorigenesis, loss of *NF1* function is linked to therapeutic resistance in multiple cancers, including resistance to RAF inhibitors in melanoma [9], to retinoic acid in neuroblastoma [10], to EGFR inhibitors in lung cancer [11], and to endocrine therapy in breast cancer [12]. However, the *NF1* locus spans approximately 280 kb and comprises 58 exons, making it one of the largest genes in the human genome. Reported pathogenic variants are distributed throughout the gene without well-defined mutational hotspots. Together, these features make missense variants in *NF1* especially difficult to interpret, leaving a high VUS burden for this gene [2,3].

To date, the GAP-related domain (GRD) is the only well-established functional domain of neurofibromin [5,13,14,15]. Functional studies of *NF1* variants, especially missense variants, have concentrated on this region, leaving variants outside the GRD largely uncharacterized [16,17]. This bias reflects both technical and biological constraints. The full-length *NF1* cDNA (~8.5 kb) is toxic and unstable in *Escherichia coli,* whereas the short GRD fragment is easily cloned and overexpressed [18]. Moreover, the GRD alone is sufficient to recapitulate neurofibromin’s RAS-GAP activity [19,20,21]. GRD overexpression thus became the standard surrogate assay, yet it cannot evaluate the majority of variants, which lie outside the GRD. CRISPR editing now allows these variants to be studied in their endogenous context. However, homology-directed repair (HDR) editing is inefficient, and base editing introduces bystander edits that preclude precise single-variant modeling [22]. As a result, the functional consequences of variants outside the GRD remain largely unresolved.

Prime editing addresses these limitations by introducing precise nucleotide substitutions directly at the endogenous locus without double-strand breaks or donor templates required for HDR [23], thereby avoiding the low efficiency of that pathway [24]. It also avoids the bystander edits introduced by base editing, so that the phenotype can be attributed to the intended substitution [25]. Moreover, because the variant is expressed from its native promoter, edited cells produce neurofibromin at physiological levels, avoiding the artifacts of overexpression and ensuring that the functional effect reflects the variant itself rather than altered protein dosage. Structural biology offers a complementary advance. High-resolution cryo-electron microscopy (cryo-EM) structures of full-length human neurofibromin reveal that it functions as a homodimer [26,27,28,29]. They also provide the first experimentally determined models for regions outside the GRD, which had been accessible only by computational prediction. This offers an accurate basis for mapping non-GRD variants and interpreting their functional consequences. Together, these advances allow non-GRD missense variants to be introduced precisely at the endogenous locus and interpreted within a high-resolution structural framework.

Here, we functionally characterized *NF1* c.6394T>C (p.Ser2132Pro), a non-GRD missense variant currently classified as a VUS in ClinVar. Using prime editing, we introduced the variant at the endogenous *NF1* locus in HEK293T and A375 cells and assessed its effect on neurofibromin function. These functional data were complemented by population-frequency and in silico prediction. The combined analysis indicates that p.Ser2132Pro impairs neurofibromin function and sustains RAS-MAPK signaling under BRAF inhibition in melanoma cells. Interpreted against the ACMG/AMP criteria, these findings support a likely pathogenic interpretation of the variant and illustrate a targeted strategy for resolving *NF1* variants beyond the GRD.

## 2. Materials and Methods

### 2.1. Variant Nomenclature and Population Frequency Annotation

The *NF1* c.6394T>C variant was analyzed on the MANE Select transcript NM_001042492.3 (p.Ser2132Pro, NP_001035957.1) and is reported here together with the MANE Plus Clinical transcript NM_000267.4:c.6331T>C (p.Ser2111Pro, NP_000258.1). Both annotations refer to the identical genomic change, NC_000017.11:g.31336881T>C (GRCh38, SPDI NC_000017.11:31336880:T:C). The 21-residue difference in codon numbering reflects inclusion of the alternatively spliced exon 23a in NM_001042492.3. The variant was identified from ClinVar as a missense variant of uncertain significance (VCV001422558.8); at the time of access the record contained no publicly available functional evidence. Variant annotation was performed with Ensembl VEP (version 111, EMBL-EBI, Hinxton, Cambridgeshire, UK) [30]. Population frequency was assessed in gnomAD v4.1 [31], and allele count, allele number, allele frequency, GroupMax filtering allele frequency, and locus-level coverage were reviewed where available. Unless otherwise specified, all database queries were last accessed on 11 June 2026.

### 2.2. Evolutionary Conservation Analysis

Evolutionary conservation at the NF1 codon 2132 position was evaluated using quantitative genomic conservation scores and qualitative cross-species amino acid alignment. PhyloP 100-way vertebrate, PhastCons 100-way vertebrate, and GERP++ RS scores were retrieved from dbNSFP v5.3.1 [32]. To visualize local amino acid conservation, neurofibromin protein sequences were downloaded from UniProtKB (UniProt Consortium) [33] for representative orthologs spanning mammals, birds, amphibians, fish, and an invertebrate outgroup. The analyzed sequences included human neurofibromin (P21359), mouse neurofibromin (Q04690), dog neurofibromin (A0A8C0NR24), chicken neurofibromin (A0A8V1ADH8), *Xenopus tropicalis* neurofibromin (A0A6I8SBJ8), zebrafish neurofibromin (A0A8M9PLQ0), and *Drosophila melanogaster* neurofibromin (O01397). Multiple sequence alignment was performed using the NCBI COBALT alignment tool (National Center for Biotechnology Information, Bethesda, MD, USA) [34]. The local aligned region surrounding the residue corresponding to *NF1* p.Ser2132Pro was extracted for visualization. Because *NF1* isoforms and database entries may differ in residue numbering, the aligned residue was mapped to the clinical reference sequence used for variant annotation. Conservation was assessed qualitatively by comparing the amino acid present at the aligned position across the selected orthologous neurofibromin sequences.

### 2.3. In Silico Pathogenicity Prediction

Computational pathogenicity predictions were obtained for *NF1* c.6394T>C (p.Ser2132Pro). The AlphaMissense score was retrieved from the AlphaMissense database [35]. The REVEL score and SpliceAI masked Δmax (the maximum delta score after filtering predictions proximal to existing splice sites) were retrieved from the UCSC Genome Browser (GRCh38/hg38 assembly, University of California, Santa Cruz, CA, USA) [36] to assess missense pathogenicity and potential splicing impact, respectively.

### 2.4. Structural Modeling and Analysis

The structural context of Ser2132 was analyzed using the cryo-EM structure of full-length human neurofibromin (PDB: 7PGR) [26]. The PDB structure (2839 aa) corresponds to isoform 2 (NM_001042492.3), and residue numbering is therefore consistent with the clinical annotation. The p.Ser2132Pro mutant structure was generated using CHARMM-GUI (Lehigh University, Bethlehem, PA, USA) [37] and visualized in PyMOL v2.6.0 (Schrödinger, LLC, New York, NY, USA). Protein stability changes upon substitution were predicted using FoldX v5.1 (Centre for Genomic Regulation, Barcelona, Spain) [38]. Relative solvent accessibility (RSA) of the wild-type residue at position 2132 was predicted using NACCESS v2.1.1 (University College London, London, UK). Interatomic contacts at the Ser2132 site were analyzed in both wild-type and mutant structures using Arpeggio (University of Cambridge, Cambridge, UK) [39].

### 2.5. Cell Lines and Culture Conditions

A375 human melanoma cells (SCSP-533) were obtained from the National Collection of Authenticated Cell Cultures, Chinese Academy of Sciences and maintained in Dulbecco’s Modified Eagle’s Medium (DMEM) supplemented with 10% fetal bovine serum (FBS), 1% penicillin-streptomycin, 1 mM sodium pyruvate, and 1% GlutaMAX supplement. HEK293T cells were obtained from the Cell Resource Center, Peking Union Medical College (National Science and Technology Infrastructure, National Biomedical Cell-Line Resource, NSTI-BMCR; http://cellresource.cn; accessed on 11 June 2026) and cultured in DMEM supplemented with 10% FBS and 1% penicillin-streptomycin. All cells were maintained at 37 °C in a humidified atmosphere containing 5% CO_2_ and were routinely tested for mycoplasma contamination.

### 2.6. Prime Editing of Endogenous NF1 Variants and Genotype Validation

The *NF1* missense variants c.6394T>C (p.Ser2132Pro) and c.3827G>A (p.Arg1276Gln) were individually introduced into the endogenous *NF1* locus of HEK293T and A375 cells by prime editing. Prime editing guide RNAs (pegRNAs) were designed using PRIDICT2.0 [40], and cloned into the pU6-tevopreq1-GG-acceptor vector (Addgene, 174038). The pCMV-PE7 prime editor plasmid (Addgene, 214812) was co-transfected with the pegRNA construct and a nicking sgRNA targeting the non-edited strand to enhance editing efficiency. Transfections were performed using Lipofectamine 3000 (Thermo Fisher Scientific, Waltham, MA, USA) according to the manufacturer’s instructions. Edited clones were selected by single-cell sorting, expanded, and genotyped by Sanger sequencing of the target locus. A single-cell clone confirmed to carry the intended homozygous genotype was used for downstream experiments for each cell line. A genotype-confirmed single-cell clone derived from cells transfected with a non-targeting pegRNA (NT) served as the matched control in all experiments. Sanger sequencing traces were analyzed using BEAT [41]. Independent replicates were defined as independent passages and independent lysate preparations of the same edited cell clone. Sequences of all pegRNAs and PCR primers are listed in Table S1.

### 2.7. Base Editing of Endogenous NF1 Variants and Genotype Validation

The *NF1* c.6394T>C (p.Ser2132Pro) variant was introduced into the endogenous locus of A375 cells by adenine base editing. The sgRNA was cloned into the lentiGuide-Puro-optimized vector [25]. A375 cells were co-transfected with the ABE8e-NL-SpRY-GFP [25] editor vector and the sgRNA construct using Lipofectamine 3000 (Thermo Fisher Scientific) following the manufacturer’s instructions. At 24 h post-transfection, GFP-positive cells were isolated by fluorescence-activated cell sorting. At 48 h post-transfection, puromycin selection was initiated to enrich for cells carrying the sgRNA construct. The resulting cell pool was used for downstream experiments. Genomic DNA was extracted, and the target region was amplified by PCR and analyzed by Sanger sequencing. Editing efficiency was quantified with BEAT [41]. Independent replicates were defined as independent passages and independent lysate preparations of the same edited cell pool. Sequences of all sgRNAs and PCR primers are listed in Table S1.

### 2.8. Western Blot Analysis and Densitometric Quantification

Cells were lysed in RIPA lysis buffer (Beyotime Biotechnology, Shanghai, China, P0013C) supplemented with protease inhibitor cocktail (Roche, Basel, Switzerland, 04693132001 and 04906837001). Protein concentration was determined by BCA assay (Thermo Fisher Scientific). Equal amounts of protein (30 µg) were resolved by SDS-PAGE on 10–12% LabPAGE precast gels (Lablead, Beijing, China) and transferred to PVDF membranes (Millipore, Burlington, MA, USA). Membranes were blocked with 3% BSA in TBST for 1 h at room temperature and incubated with primary antibodies overnight at 4 °C. The following primary antibodies were used at a 1:1000 dilution unless otherwise stated: anti-Neurofibromin (Abcam, Cambridge, UK, ab17963, immunogen amino acids 2700–2839; Abcam, ab128054, immunogen amino acids 1501–1650), pan-RAS (Thermo Fisher Scientific, MA1-012), phospho-ERK1/2 (Thr202/Tyr204) (Cell Signaling Technology, Danvers, MA, USA, 4370), total ERK1/2 (Cell Signaling Technology, 4695), phospho-MEK1/2 (Ser217/221) (Cell Signaling Technology, 9154), total MEK1/2 (Cell Signaling Technology, 8727), beta-actin (Cell Signaling Technology, 4970), and GAPDH (Proteintech, Rosemont, IL, USA, HRP-60004). After washing with TBST, membranes were incubated with HRP-conjugated secondary antibodies (EASYBIO, Beijing, China, BE0101 or BE0141, 1:5000) for 1 h at room temperature. Signals were detected using enhanced chemiluminescence (ECL) reagent and imaged on a ChemiDoc imaging system (Bio-Rad, Hercules, CA, USA). Band intensities were quantified by densitometry using Image Lab software v6.1 (Bio-Rad, Hercules, CA, USA). Neurofibromin abundance was normalized to the loading control beta-actin and expressed relative to the NT control. Phospho-MEK and phospho-ERK signals were normalized to their respective total protein and expressed relative to the NT control. For the base-edited A375 cell pools, neurofibromin abundance was normalized to GAPDH as the loading control.

### 2.9. RT-qPCR Assay

Total RNA was extracted using the RNA-Quick Purification Kit (ES Science, Shanghai, China, RN001) according to the manufacturer’s instructions. RNA concentration and purity were assessed by NanoDrop (Thermo Fisher Scientific, Waltham, MA, USA). Complementary DNA (cDNA) was synthesized from 3 µg of total RNA using the RevertAid First Strand cDNA Synthesis Kit (Thermo Fisher Scientific, K1622) according to the manufacturer’s protocol. Gene expression was quantified by RT-qPCR using TransStart qPCR SuperMix (TransGen Biotech, Beijing, China, AQ631-04) on a QuantStudio 5 real-time PCR system (Applied Biosystems, Foster City, CA, USA). Reactions were performed in triplicate in a total volume of 10 µL. Primer sequences are listed in Table S1. Relative mRNA expression was calculated using the ΔΔCt method with *GAPDH* as the reference gene. Results are expressed as fold change relative to the non-targeting (NT) control.

### 2.10. RAS-GTP Pull-Down Assay

Active (GTP-bound) RAS levels were measured using the Active Ras Pull-Down and Detection Kit (Thermo Fisher Scientific, 16117) according to the manufacturer’s instructions. Briefly, cells were lysed in the provided lysis/binding/wash buffer supplemented with protease inhibitor cocktail (Roche, 04693132001 and 04906837001). Clarified lysates (500 µg total protein) were incubated with glutathione resin pre-loaded with GST-tagged RAF1 RAS-binding domain (RBD) for 1 h at 4 °C with gentle rotation. Resin was washed three times, and bound proteins were eluted in SDS sample buffer and analyzed by Western blot using the pan-RAS antibody (Thermo Fisher Scientific, MA1-012). Total RAS in whole-cell lysate was used as a loading control. For steady-state RAS-GTP measurements in A375 cells, cells were seeded at equal density in 6 cm dishes 24 h before collection. For serum-stimulation time-course experiments, HEK293T cells were serum-starved for 14 h in DMEM without FBS, then stimulated with 1% FBS for 0, 10, or 30 min before lysis. RAS-GTP levels were normalized to total RAS and expressed relative to the NT control at each time point.

### 2.11. PLX4032 Treatment and MAPK Pathway Analysis

PLX4032 (vemurafenib; Selleckchem, Houston, TX, USA, S1267) was dissolved in dimethyl sulfoxide (DMSO) to prepare a 100 mM stock solution and stored at −20 °C. A375 cells were treated with PLX4032 at 1 µM for 16 h. DMSO at an equivalent volume was used as a vehicle control. Following treatment, cells were lysed and analyzed by Western blot for phospho-MEK1/2 (Ser217/221), total MEK1/2, phospho-ERK1/2 (Thr202/Tyr204), and total ERK1/2 as described in Section 2.8.

### 2.12. ACMG/AMP Variant Classification

Pathogenicity was assessed under the 2015 ACMG/AMP framework operationalized as the Bayesian point-based system [42,43], incorporating relevant ClinGen Sequence Variant Interpretation (SVI) recommendations. Criterion strength modifications followed the ClinGen SVI nomenclature, in which the original criterion code is appended with an underscore and the modified strength level [44]. No ClinGen-approved *NF1*-specific specifications were available. The ClinGen Neurofibromatosis and Schwannomatosis Variant Curation Expert Panel (VCEP) had submitted pilot rules, but these had not received ClinGen approval at the time of analysis, so the general framework was applied. Computational and splicing evidence was applied following ClinGen SVI recommendations [45,46]. Given the strong correlation among missense pathogenicity predictors, computational evidence was not derived by counting concordant tools. Instead, a single calibrated predictor was pre-specified as the PP3 anchor and applied using ClinGen-calibrated evidence-strength thresholds rather than developer-recommended cut-offs. AlphaMissense was selected as the PP3 anchor based on its ClinGen-calibrated evidence-strength thresholds and its incorporation of protein structural information, which provides a methodological basis distinct from sequence-based meta-predictors. Splicing impact was assessed with SpliceAI using the ClinGen SVI Splicing Subgroup generic thresholds (PP3 ≥ 0.2, BP4 ≤ 0.1). Functional evidence (PS3) was evaluated following ClinGen SVI recommendations [47]. Endogenous prime-edited cellular assays were benchmarked against a ClinVar-pathogenic comparator (p.Arg1276Gln) and NT controls. The applied strength of PS3 was determined by the number of available validation controls. According to ClinGen SVI guidance, assays with 10 or fewer validation controls may support PS3 at the Supporting level. Population evidence (PM2) was assessed using the GroupMax filtering allele frequency in gnomAD v4.1, and applied at Supporting strength per ClinGen SVI [48]. PP2 was deliberately not applied, to avoid double-counting the missense-impact signal already captured by the calibrated PP3 line. PP1, PS2, and PP4 were not applicable because segregation, de novo, and detailed proband-phenotype data were unavailable. Benign criteria (BS1, BS2, BP4, BP7) were also evaluated. Whether each criterion applied was determined by the corresponding population frequency, computational, and splicing evidence according to ACMG/AMP and ClinGen SVI specifications. Evidence was combined under both the categorical and point-based rules.

### 2.13. Statistical Analysis

All quantitative experiments were performed in at least three independent replicates. Data are presented as mean ± standard deviation (SD). Statistical comparisons between two groups were performed using two-tailed unpaired Student’s *t*-tests; exact *p* values are provided in the Supplementary Tables where applicable. A *p* value < 0.05 was considered statistically significant. Statistical analyses were performed using GraphPad Prism v10.0 (GraphPad Software, Boston, MA, USA).

## 3. Results

### 3.1. Identification and In Silico Characterization of NF1 c.6394T>C (p.Ser2132Pro)

**Figure 1:**
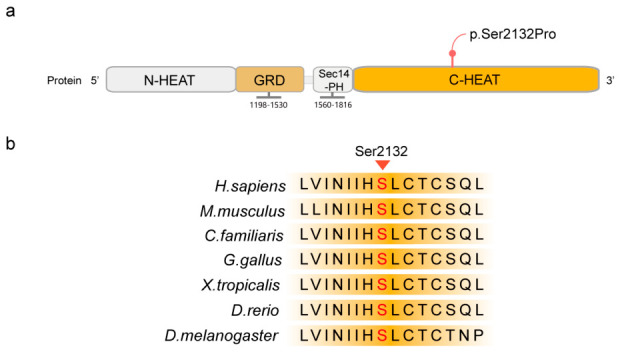
Location within neurofibromin and evolutionary conservation of the *NF1* p.Ser2132Pro variant. (**a**) Schematic representation of human neurofibromin (drawn not to scale), showing the N-terminal HEAT-repeat region (N-HEAT; residues 1–1197, gray), the GTPase-activating protein (GAP)-related domain (GRD; residues 1198–1530, yellow), the Sec14-pleckstrin homology (Sec14-PH) module (residues 1560–1816, gray), and the C-terminal HEAT-repeat region (C-HEAT; residues 1817–2839, orange). The p.Ser2132Pro substitution is located in the C-terminal region of the protein. (**b**) Multiple sequence alignment of the neurofibromin region surrounding human Ser2132 in representative vertebrate species (*Homo sapiens*, *Mus musculus*, *Canis familiaris*, *Gallus gallus*, *X. tropicalis*, and *Danio rerio*) and the invertebrate outgroup *D. melanogaster*. The serine residue at position 2132 (orange highlight; red arrowhead) is highly conserved.

*NF1* c.6394T>C (p.Ser2132Pro) is a missense variant in exon 42 that replaces serine with proline at codon 2132, within the C-terminal region of neurofibromin distal to the GAP-related domain (Figure 1a). This variant is currently classified as a VUS in ClinVar and is absent from gnomAD (mean exome coverage 35.62×, with 100% of samples covered at 20× at this position). The Ser2132 residue was conserved across all examined species, including the invertebrate outgroup *D. melanogaster* (Figure 1b), and all three conservation metrics exceeded their predefined thresholds (Table 1).

Calibrated missense prediction models and a splice-impact predictor were then used to assess the likely consequence of the substitution (Table 2). AlphaMissense yielded a score of 0.9997, above the predefined high-confidence threshold for a deleterious classification. REVEL provided concordant support for a damaging missense effect, with a score of 0.914. In contrast, SpliceAI did not predict a splice-altering effect (masked Δmax = 0.06), falling within the non-spliceogenic range (≤0.10). Together, these computational results support interpreting p.Ser2132Pro primarily at the protein level rather than through a splice-mediated mechanism.

### 3.2. p.Ser2132Pro Reduces Neurofibromin Abundance

**Figure 2:**
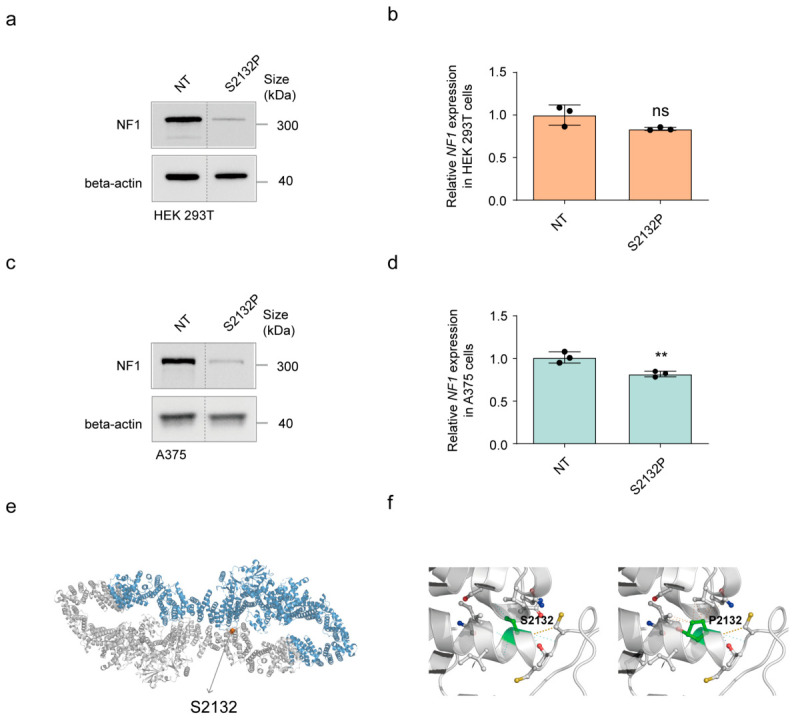
Effect of the *NF1* p.Ser2132Pro variant on protein abundance and transcript levels, and its structural context. (**a**,**c**) Representative Western blots showing NF1 and beta-actin in HEK293T cells (**a**) and A375 cells (**c**) harboring either a non-targeting control (NT) or the p.Ser2132Pro (S2132P) variant generated by prime editing. Dashed lines indicate lane boundaries from the same blot. Molecular weight markers are shown in kilodaltons (kDa). Blots are representative of three independent experiments. Densitometric quantification is provided in Figure S2, and source densitometric values and normalized data are provided in Table S2. (**b**,**d**) Relative *NF1* mRNA levels quantified by RT-qPCR in HEK293T cells (**b**) and A375 cells (**d**) carrying the NT control or the p.Ser2132Pro (S2132P) variant. Expression was normalized to *GAPDH* and is shown relative to NT. Bars represent the mean ± standard deviation (SD), and individual data points denote three independent replicates. ns, not significant; ** *p* < 0.01 versus NT, two-tailed Student’s *t*-test. Source and normalized expression data are provided in Table S3. (**e**) Ribbon representation of the full-length human neurofibromin homodimer structure (PDB: 7PGR). One protomer is colored blue and the other gray. The Ser2132 (S2132) residue is shown as orange dots and indicated by a gray arrow. (**f**) Close-up views of the local structural environment surrounding residue 2132 in the wild-type protein (**left**, S2132) and the in silico-modeled mutant protein (**right**, P2132). The residue of interest is shown in green, and neighboring residues are displayed as sticks. Dashed interaction lines indicate hydrogen-bond-associated contacts (marine blue), polar contacts (cyan), hydrophobic contacts (yellow-orange), and weak-polar/van der Waals contacts (red). The mutant model was generated using CHARMM-GUI and visualized in PyMOL.

To test the functional consequences of this variant at the native locus, we used prime editing to introduce p.Ser2132Pro at the endogenous *NF1* locus, with the intended edit confirmed by Sanger sequencing (Figure S1). In HEK293T cells, neurofibromin abundance was reduced to 5.5% of NT controls (*p* = 0.0026, Figure 2a and Figure S2a), whereas *NF1* mRNA levels were not significantly altered (*p* = 0.0791, Figure 2b). In A375 cells, neurofibromin abundance was similarly reduced to 12.2% of NT controls (*p* = 0.0024, Figure 2c and Figure S2b), whereas *NF1* transcript levels were modestly reduced to 80.8% (*p* = 0.0099, Figure 2d). As orthogonal validation, we introduced the same substitution in A375 cells by adenine base editing with a 72% editing efficiency (Figure S3a), with a synonymous bystander edit at the third position of the same codon (Figure S3b). Neurofibromin abundance was again reduced to 24% of WT controls (*p* = 0.0039, Figure S3c,d). This reduction was independently confirmed using a second anti-neurofibromin antibody recognizing a distant epitope, which likewise showed reduced neurofibromin abundance to 35% of NT controls (*p* = 0.0295, Figure S3e,f). The disproportionate reduction in protein relative to transcript suggests that the reduction in neurofibromin occurs at least in part post-transcriptionally.

To examine the structural context of Ser2132, we mapped the residue onto the full-length human neurofibromin homodimer cryo-EM structure. Ser2132 lies in a buried α-helical segment of the C-HEAT region, adjacent to the dimer core (Figure 2e, Table 3). Substitution with proline, a helix-breaking residue [49], is predicted to disrupt local helical packing at this buried site. Consistent with this prediction, FoldX predicted destabilization across all examined cryo-EM structures, and Arpeggio analysis supported local remodeling of the interaction network, characterized by loss of polar interactions and increased van der Waals clashes around the substituted proline (Figure 2f, Table 3 and Table 4).

### 3.3. p.Ser2132Pro Impairs Termination of RAS Signaling in HEK293T Cells

We then examined whether the reduced neurofibromin abundance in p.Ser2132Pro cells was accompanied by impaired RAS-GTP signal termination in HEK293T cells. NT cells served as the negative control, and p.Arg1276Gln cells as a pathogenic comparator, because this variant disrupts the neurofibromin arginine finger essential for RAS-GAP activity [14,50]. Introduction of p.Arg1276Gln at the endogenous *NF1* locus was confirmed by Sanger sequencing (Figure S4). After serum starvation and low-serum stimulation, active RAS-GTP was captured by GST-RBD pull-down.

**Figure 3:**
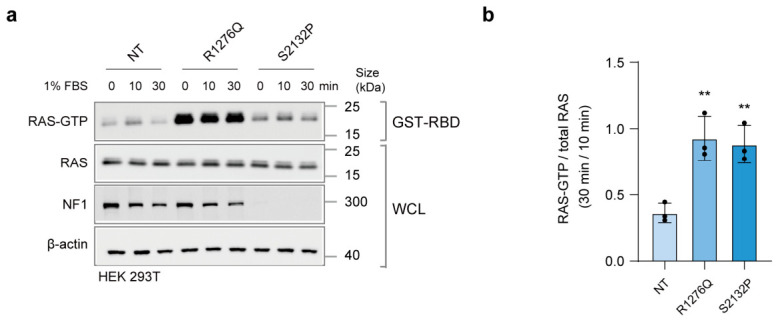
The *NF1* p.Ser2132Pro variant impairs termination of RAS activation, sustaining cellular RAS-GTP signaling in HEK293T cells. (**a**) Representative Western blot of GST-RBD pull-down assays in serum-starved HEK293T cells harboring NT, p.Arg1276Gln (R1276Q), or p.Ser2132Pro (S2132P), followed by stimulation with 1% FBS for the indicated times. RAS-GTP was detected in the GST-RBD pull-down fraction, and total RAS, NF1, and beta-actin were detected in whole-cell lysates (WCL). Molecular weights are indicated in kilodaltons (kDa). (**b**) Quantification of RAS-GTP normalized to total RAS, expressed as the ratio of the normalized signal at 30 min relative to that at 10 min for each cell line. Bars represent the mean ± standard deviation (SD), and individual data points denote three independent replicates. ** *p* < 0.01 versus NT, two-tailed Student’s *t*-test. Source densitometric values and normalized data are provided in Table S5.

Under these conditions, NT control cells displayed the expected kinetics of RAS-GTP signal termination, with levels rising upon stimulation and declining by 30 min (Figure 3a). In contrast, both p.Ser2132Pro and p.Arg1276Gln cells showed sustained RAS-GTP levels, with impaired decay between 10 and 30 min (Figure 3a). When quantified as the 30 min/10 min RAS-GTP ratio, termination was significantly impaired in both variants relative to NT. The ratio was 0.88 in p.Ser2132Pro cells (*p* = 0.0049) and 0.93 in p.Arg1276Gln cells (*p* = 0.0058), compared with 0.36 in NT (Figure 3b). Despite this shared functional defect, the two variants differed in neurofibromin abundance. p.Ser2132Pro cells showed reduced neurofibromin, whereas p.Arg1276Gln cells retained normal levels (Figure 3a).

### 3.4. p.Ser2132Pro Impairs NF1 RAS-GAP Activity in A375 Melanoma Cells

To further support the functional evidence for RAS-GAP impairment, we examined p.Ser2132Pro in a second cellular background using A375 melanoma cells, which harbor an endogenous BRAF V600E mutation and represent an established model in which *NF1* loss-of-function confers resistance to RAF inhibitors [9,51]. We additionally introduced p.Arg1276Gln at the endogenous *NF1* locus in A375 cells by prime editing and the intended edit was confirmed by Sanger sequencing (Figure S5).

**Figure 4:**
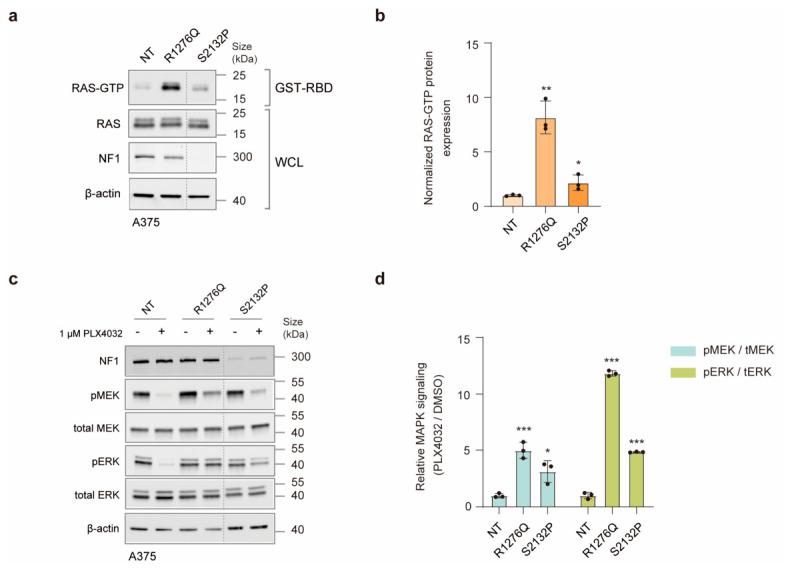
The *NF1* p.Ser2132Pro variant elevates steady-state RAS-GTP and attenuates PLX4032-mediated suppression of MAPK signaling in A375 melanoma cells. (**a**) Representative Western blot of GST-RBD pull-down assays in A375 cells expressing NT, p.Arg1276Gln (R1276Q), or p.Ser2132Pro (S2132P) under steady-state conditions. RAS-GTP was detected in the GST-RBD pull-down fraction, and total RAS, NF1, and beta-actin were detected in whole-cell lysates (WCL). Molecular weights are indicated in kilodaltons (kDa). (**b**) Quantification of steady-state RAS-GTP normalized to total RAS and expressed relative to NT. (**c**) Representative Western blot of NF1, phospho-MEK (pMEK), total MEK (tMEK), phospho-ERK (pERK), total ERK (tERK), and beta-actin in cells treated with DMSO vehicle (−) or 1 µM PLX4032 (+) for 16 h. (**d**) Quantification of pMEK/tMEK and pERK/tERK ratios after PLX4032 treatment, expressed relative to the corresponding DMSO-treated control within each genotype. Dashed lines indicate lane boundaries from the same blot. Bars in (**b**,**d**) represent the mean ± standard deviation (SD), and individual data points denote three independent replicates. * *p* < 0.05; ** *p* < 0.01; *** *p* < 0.001 versus NT, two-tailed Student’s *t*-test. Source densitometric values and normalized data are provided in Table S6.

Under steady-state conditions, RAS-GTP levels were 2.17-fold higher in p.Ser2132Pro cells (*p* = 0.048) and 8.16-fold higher in p.Arg1276Gln cells (*p* = 0.0012) relative to NT (Figure 4a,b), indicating impaired neurofibromin-mediated negative regulation of RAS. We then examined whether elevated RAS-GTP was accompanied by MAPK pathway activation under RAF inhibition. In the DMSO group, pMEK and pERK levels were comparable across all three lines (Figure 4c), consistent with strong ERK-dependent negative feedback from constitutive BRAF V600E signaling, which masks the impact of elevated RAS-GTP on downstream output [9].

Following PLX4032 treatment, residual MAPK pathway activity was significantly elevated in both variant cell lines. Residual pMEK was 3.1-fold higher in p.Ser2132Pro cells (*p* = 0.020) and 5.0-fold higher in p.Arg1276Gln cells (*p* = 7.2 × 10^−4^) relative to NT, with corresponding residual pERK 4.8-fold higher in p.Ser2132Pro cells (*p* = 1.9 × 10^−5^) and 11.8-fold higher in p.Arg1276Gln cells (*p* = 8.7 × 10^−7^) (Figure 4c,d). The elevated residual pMEK and pERK in p.Ser2132Pro cells following RAF inhibition are consistent with the elevated steady-state RAS-GTP levels in the same cells, together providing functional evidence of impaired *NF1* RAS-GAP activity.

### 3.5. ACMG/AMP-Based Evaluation Supports a Likely Pathogenic Interpretation of NF1 c.6394T>C

*NF1* c.6394T>C (p.Ser2132Pro) was classified under the ACMG/AMP framework operationalized as the Bayesian point-based system [43], with the computational, population, and functional evidence summarized in Table 5.

Computational evidence was applied as PP3_Strong (+4 points), based on an AlphaMissense score of 0.9997 within the calibrated Strong interval. REVEL (0.914) was directionally concordant. PP2 was not applied (Method). Population evidence was applied as PM2_Supporting (+1 point), as the variant is absent from gnomAD at an adequately covered position.

Functional evidence was applied as PS3_Supporting (+1 point). *NF1*-associated disease is driven by loss of neurofibromin RAS-GAP function, and the assays employed here directly measure this molecular function. Variants were introduced by prime editing at the endogenous *NF1* locus to preserve native regulatory context, and RAS-GAP function was evaluated across two independent cellular backgrounds using complementary functional readouts, with the ClinVar variant p.Arg1276Gln serving as a pathogenic control. Specific functional readouts are summarized in Table 5. Strength was applied at Supporting, reflecting the limited number of validation controls (Method).

Together, PP3_Strong (+4), PM2_Supporting (+1) and PS3_Supporting (+1) yielded a total of 6 points, meeting the threshold for Likely Pathogenic (6–9 points). No benign criteria applied. The available evidence therefore supports a likely pathogenic interpretation of *NF1* c.6394T>C (p.Ser2132Pro).

## 4. Discussion

In this study, we used prime editing to introduce the *NF1* missense variant c.6394T>C (p.Ser2132Pro) into its endogenous locus and evaluated its functional consequences. The variant disproportionately reduced neurofibromin protein abundance relative to the modest reduction in *NF1* mRNA. Across two cellular contexts, p.Ser2132Pro compromised the RAS-GAP activity of neurofibromin, as reflected by impaired signal termination after serum stimulation in HEK293T cells and elevated steady-state RAS-GTP with sustained MAPK activity under RAF inhibition in A375 cells. These findings are consistent with the loss-of-function effect observed for the ClinVar-classified pathogenic control p.Arg1276Gln. Together with the absence of this variant from population databases and calibrated computational predictions, these functional data support a likely pathogenic interpretation under the ACMG/AMP framework.

The architecture of neurofibromin also provides a structural framework for understanding how variants outside the catalytic GRD may affect protein function. Cryo-EM studies have shown that neurofibromin forms a large homodimeric scaffold with a lemniscate-like architecture composed primarily of N- and C-terminal HEAT-repeat domains [26,27,28,29]. Several C-HEAT variants, including L1834R, N1840K, and L2104R, have been reported to reduce protein abundance or thermal stability, with L1834R and L2104R also altering the dimer–monomer equilibrium [27]. Ser2132 is located within a packed α-helical segment of the C-HEAT region, and substitution with proline is predicted to alter the local structural environment, potentially contributing to the reduced neurofibromin abundance observed in our cellular assays. However, computational predictions alone cannot establish the mechanisms responsible for the reduced protein abundance. Direct structural, biophysical, and protein-turnover studies will be required to define the underlying mechanism.

The comparison between p.Ser2132Pro and p.Arg1276Gln reveals two distinct mechanisms of *NF1* functional impairment. Whereas p.Arg1276Gln disrupts the conserved arginine finger within the GRD and impairs GAP catalytic activity [50,52], p.Ser2132Pro primarily reduces neurofibromin protein levels. Despite a reduction of 88–95% in neurofibromin abundance, p.Ser2132Pro did not match the severity of p.Arg1276Gln, suggesting that the residual mutant neurofibromin may retain GAP activity to partially sustain RAS regulation, although its catalytic activity was not directly measured. This distinction would have been difficult to resolve with overexpression-based assays, which may mask abundance defects by producing non-physiological protein concentrations and thereby overestimate residual neurofibromin function [42,47]. Our results therefore highlight the importance of endogenous-locus editing for evaluating variants that reduce protein abundance. These findings are supported by the concordant molecular phenotypes observed across two independently edited cellular backgrounds and by orthogonal validation with adenine base editing in unselected A375 cell pools, which together reduce the likelihood that the observed phenotype resulted from clone-specific or editing-related artifacts.

The assays used here measured *NF1*-mediated RAS regulation, a molecular function relevant to the established loss-of-function disease mechanism. The impaired RAS-GAP function observed for p.Ser2132Pro provides functional evidence supporting application of the ACMG/AMP PS3 criterion. HEK293T cells have a well-characterized genomic background and carry no reported activating mutations in the RAS pathway [53,54], providing a defined context in which RAS-GTP kinetics serve as a direct functional readout of neurofibromin GAP activity. An independent cellular context was provided by A375 melanoma cells, in which PLX4032 relieves ERK-dependent negative feedback and makes MAPK signaling more dependent on upstream RAS activity [9,55]. Under this condition, the sustained MEK–ERK phosphorylation observed in p.Ser2132Pro cells reflects impaired RAS regulation and provides an additional molecular readout of the RAS-GAP functional defect. Although the concordant findings across the two models support a molecular loss-of-function effect, neither model recapitulates the Schwann-lineage context of *NF1*-associated germline disease, and the homozygous edited genotype used here may amplify the observed functional effect relative to the heterozygous germline state. These data provide variant-level functional evidence satisfying the PS3 criterion of the ACMG/AMP framework, but do not establish clinical pathogenicity of the variant. The current classification therefore rests on the integration of functional, population, and computational evidence [47].

Several limitations should be considered. The mechanism underlying the reduced neurofibromin abundance was not directly established. Cycloheximide chase assays, MG132 treatment, and ubiquitination assays would be needed to determine whether the reduced abundance reflects accelerated proteasomal degradation, while biophysical and translational studies would be required to evaluate altered protein folding, stability, or synthesis. Genotyping was based on Sanger sequencing with BEAT-based quantification, which has limited sensitivity for detecting low-frequency indels and complex allelic mixtures, and systematic off-target profiling was not performed. Targeted amplicon deep sequencing would provide more comprehensive characterization of editing outcomes. The RAS-GTP assay captured a defined activation–attenuation interval and did not resolve the full kinetics of signal termination. In addition, the experiments were conducted in immortalized cell lines with homozygous editing, and validation in heterozygous, Schwann-lineage, or patient-derived systems would be valuable for assessing the variant in a more disease-relevant context. Finally, no patient-level evidence, including de novo occurrence or co-segregation in affected families, was available. Such genetic evidence could provide additional ACMG/AMP evidence and further refine the pathogenicity classification [47].

*NF1*-specific variant interpretation guidelines remain under development, and functional evidence for non-GRD missense variants of uncertain significance remains limited. Systematic functional studies using endogenous genome editing may facilitate the functional characterization of *NF1* variants and improve the interpretation of non-GRD missense variants of uncertain significance. Integration with orthogonal evidence, including multi-omic characterization of variant consequences, may further improve the understanding of genotype-phenotype relationships and support variant pathogenicity assessment [56].

## 5. Conclusions

We found that *NF1* c.6394T>C (p.Ser2132Pro) markedly reduced endogenous neurofibromin abundance, disproportionate to the modest reduction in *NF1* mRNA. In HEK293T cells, p.Ser2132Pro impaired termination of RAS-GTP signaling. In A375 cells, p.Ser2132Pro increased steady-state RAS-GTP and sustained MEK–ERK phosphorylation under PLX4032 treatment. These findings indicate a molecular loss-of-function effect relevant to the established disease mechanism of *NF1*. Combined with the absence of the variant from population databases and calibrated computational predictions, this functional evidence supports a likely pathogenic interpretation under the ACMG/AMP framework.

## Figures and Tables

**Table 1:** Evolutionary conservation of the NF1 Ser2132 position.

Conservation Metric	Score	Reference Threshold ^1^
PhyloP 100-way vertebrate	7.494	>1.5
PhastCons 100-way vertebrate	1.000	>0.8
GERP++ RS	5.48	>4

^1^ Reference thresholds are provided to aid interpretation and do not represent formal ACMG/AMP classification criteria.

**Table 2:** In silico predictions for *NF1* c.6394T>C (p.Ser2132Pro).

Category	Tool	Output	Threshold	Interpretation
Missense impact	AlphaMissense	0.9997	≥0.990 ^1^	Likely pathogenic
Missense impact	REVEL	0.914	[0.879, 0.931] ^1^	Damaging
Splicing impact	SpliceAI (Δmax)	0.06	≥0.20 ^2^	No predicted splicing effect

^1^ ClinGen Sequence Variant Interpretation (SVI) Working Group-calibrated threshold for PP3_Strong evidence: AlphaMissense ≥ 0.990; REVEL interval [0.879, 0.931] [45]. ^2^ SpliceAI Δmax represents the maximum raw delta score across acceptor gain, acceptor loss, donor gain, and donor loss predictions. According to ClinGen SVI Splicing Subgroup recommendations, scores ≤ 0.10 support non-spliceogenicity, scores > 0.10 but <0.20 are uninformative, and scores ≥ 0.20 support spliceogenicity [46].

**Table 3:** Predicted impact of the *NF1* p.Ser2132Pro variant on protein stability and solvent accessibility across multiple cryo-EM structures.

PDB ID	Mutation	ΔΔG ^1^	RSA ^2^
7PGR	p.Ser2132Pro	7.88	0.06, 0.06
7PGS	p.Ser2132Pro	7.22	0.05, 0.04
7PGT	p.Ser2132Pro	7.27	0.04, 0.05
7PGU	p.Ser2132Pro	9.35	0.05, 0.05
7R04 ^3^	p.Ser2132Pro	10.32	0.02, 0.04
7R03 ^3^	p.Ser2132Pro	9.23	0.04, 0.05
8E20 ^3^	p.Ser2132Pro	7.41	0.04, 0.06

^1^ ΔΔG values (kcal/mol) were predicted using FoldX; positive values indicate destabilization. ^2^ RSA, relative solvent accessibility; values represent the solvent accessibility of the wild-type residue at the corresponding position, reported for each protomer in the dimeric structure. Values < 0.20 denote buried residues. ^3^ The variant is designated p.Ser2132Pro throughout this manuscript, following NM_001042492.3. In PDB entries 7R04, 7R03 and 8E20, which were built on the alternative transcript (NM_000267.4), the same physical residue is numbered Ser2111. The equivalent residue was modeled in all structures.

**Table 4:** Key Arpeggio-predicted interatomic contacts at the *NF1* p.Ser2132Pro site ^1^.

Interaction Category	WT	p.Ser2132Pro	Change	Interpretation
Hydrogen bonds	3	1	−2	Loss of local hydrogen bonds
Polar contacts	7	2	−5	Reduced local polar contact network
VdW clash interactions	3	11	+8	Increased local steric clashes
Hydrophobic contacts	0	7	+7	Hydrophobic contacts at a polar site

^1^ Arpeggio analysis was run on PDB 7PGR. This table summarizes four major interaction categories that capture distinct local biophysical changes associated with the substitution. The complete Arpeggio-derived contact profile is provided in Table S4. WT, wild-type; VdW, van der Waals.

**Table 5:** ACMG/AMP evidence summary for *NF1* c.6394T>C (p.Ser2132Pro) ^1^.

Criterion	Strength	Evidence
PP3	Strong (+4)	AlphaMissense score of 0.9997, within the ClinGen-calibrated Strong interval (≥0.990); selected as the primary calibrated computational predictor.
PS3	Supporting (+1)	Prime-edited HEK293T and A375 cells: reduced neurofibromin abundance, impaired RAS-GTP attenuation (HEK293T), elevated steady-state RAS-GTP and sustained pMEK/pERK under PLX4032 (A375); concordant with p.Arg1276Gln control.
PM2	Supporting (+1)	Absent from gnomAD v4.1 at an adequately covered position.
Total	6 points; Likely Pathogenic	Point-based classification: 6 points, consistent with Likely Pathogenic. Categorical classification: 1 Strong + 2 Supporting, consistent with Likely Pathogenic.

^1^ Criterion strengths and point values were assigned according to ACMG/AMP guidance and the Bayesian point-based classification system. No benign criteria were applicable: SpliceAI did not support a splice-altering mechanism, and no population-frequency or functional evidence supported a benign interpretation.

## Data Availability

The data presented in this study are available within the article and its Supplementary Materials. The Supplementary Materials include Sanger sequencing validation, raw and normalized densitometry values, structural contact summaries, and pegRNA/PCR oligo sequences. Publicly available datasets analyzed in this study include ClinVar, gnomAD, UniProt, and the Protein Data Bank. Additional data are available from the corresponding author upon reasonable request.

## Supplementary Materials

The following supporting information can be downloaded at: https://www.mdpi.com/article/10.3390/genes17070838/s1, Figure S1: Sanger sequencing validation of the prime-edited *NF1* c.6394T>C (p.Ser2132Pro) variant at the endogenous locus; Figure S2: Quantification of NF1 protein abundance in prime-edited *NF1* p.Ser2132Pro cells; Figure S3: Orthogonal validation of reduced neurofibromin abundance in adenine base-edited A375 cell pools; Figure S4: Sanger sequencing validation of the prime-edited *NF1* p.Arg1276Gln variant at the endogenous locus in HEK293T cells; Figure S5: Sanger sequencing validation of the prime-edited *NF1* p.Arg1276Gln variant at the endogenous locus in A375 cells; Figure S6: Uncropped Western blot images corresponding to Figure 2, Figure 3 and Figure 4 and S3; Table S1: Sequences of oligos and guide RNAs used in this study; Table S2: Quantitative densitometry data for Western blot analyses shown in Figure S2; Table S3: Source qPCR data and normalized expression values corresponding to Figure 2b,d; Table S4: Summary of Arpeggio-derived interatomic contact profiles prediction for wild-type and the S2132P variant; Table S5: Quantitative densitometry data for Western blot analyses shown in Figure 3b; Table S6: Quantitative densitometry data for Western blot analyses shown in Figure 4b,d; Table S7: Quantitative densitometry data for Western blot analyses shown in Figure S3d,f.
