# Supplementary material for: Prime Editing-Based Functional Characterization Supports a Likely Pathogenic Interpretation of NF1 c.6394T>C (p.Ser2132Pro)
Source: Genes (Basel). 2026 Jul 21;17(7):838. doi: 10.3390/genes17070838 (PMC13410078; doi:10.3390/genes17070838)

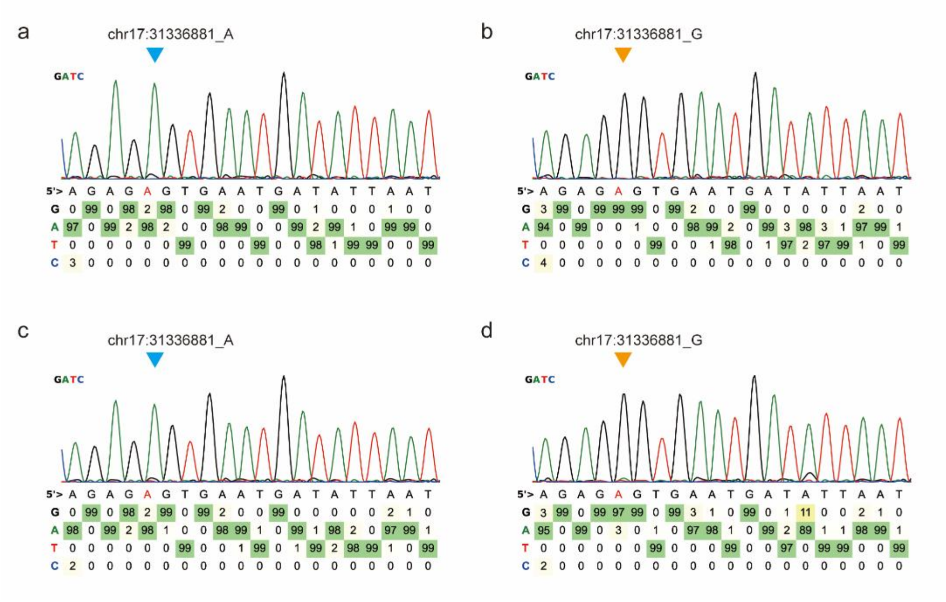

Figure S1. Sanger sequencing validation of the prime-edited *NF1* c.6394T>C (p.Ser2132Pro) variant at the endogenous locus. Representative Sanger chromatograms spanning chr17:31,336,881 (GRCh38) in non-targeting (NT) control cells (**a**,**c**) and prime-edited cells carrying the *NF1* c.6394T>C edit (**b**,**d**) are shown for HEK293T (**a**,**b**) and A375 (**c**,**d**) cells. Sequencing was performed on the antisense strand, so the targeted nucleotide reads as A in NT controls (blue arrowheads, **a**,**c**) and as G in edited cells (orange arrowheads, **b**,**d**), corresponding to the c.6394T>C substitution on the sense strand.

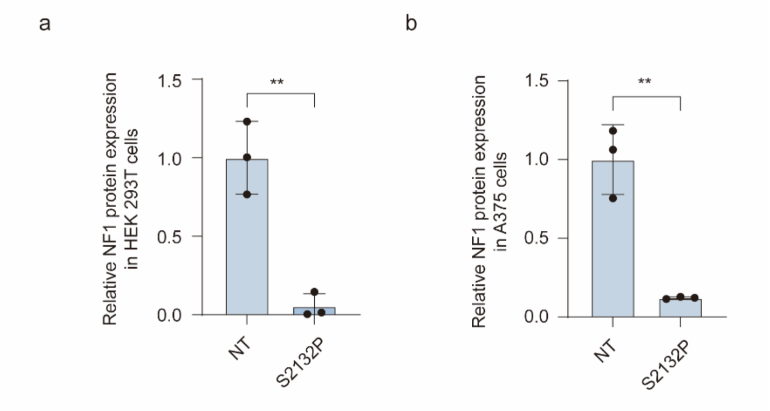

Figure S2. Quantification of NF1 protein abundance in prime-edited *NF1* p.Ser2132Pro cells. Densitometric quantification of NF1 protein bands from the Western blots shown in Figure 2a,c in (**a**) HEK293T and (**b**) A375 cells comparing NT controls with *NF1* p.Ser2132Pro (S2132P) prime-edited cells. NF1 signal was normalized to β-actin and expressed relative to the mean of the NT group within each

cell line. Bars in (**a**) and (**b**) represent the mean ± standard deviation (SD), with individual data points (filled circles) denoting three independent replicates. Raw densitometry values are provided in Table S2. Statistical significance was assessed by two-tailed unpaired Student's *t*-test; ** $p < 0.01$.

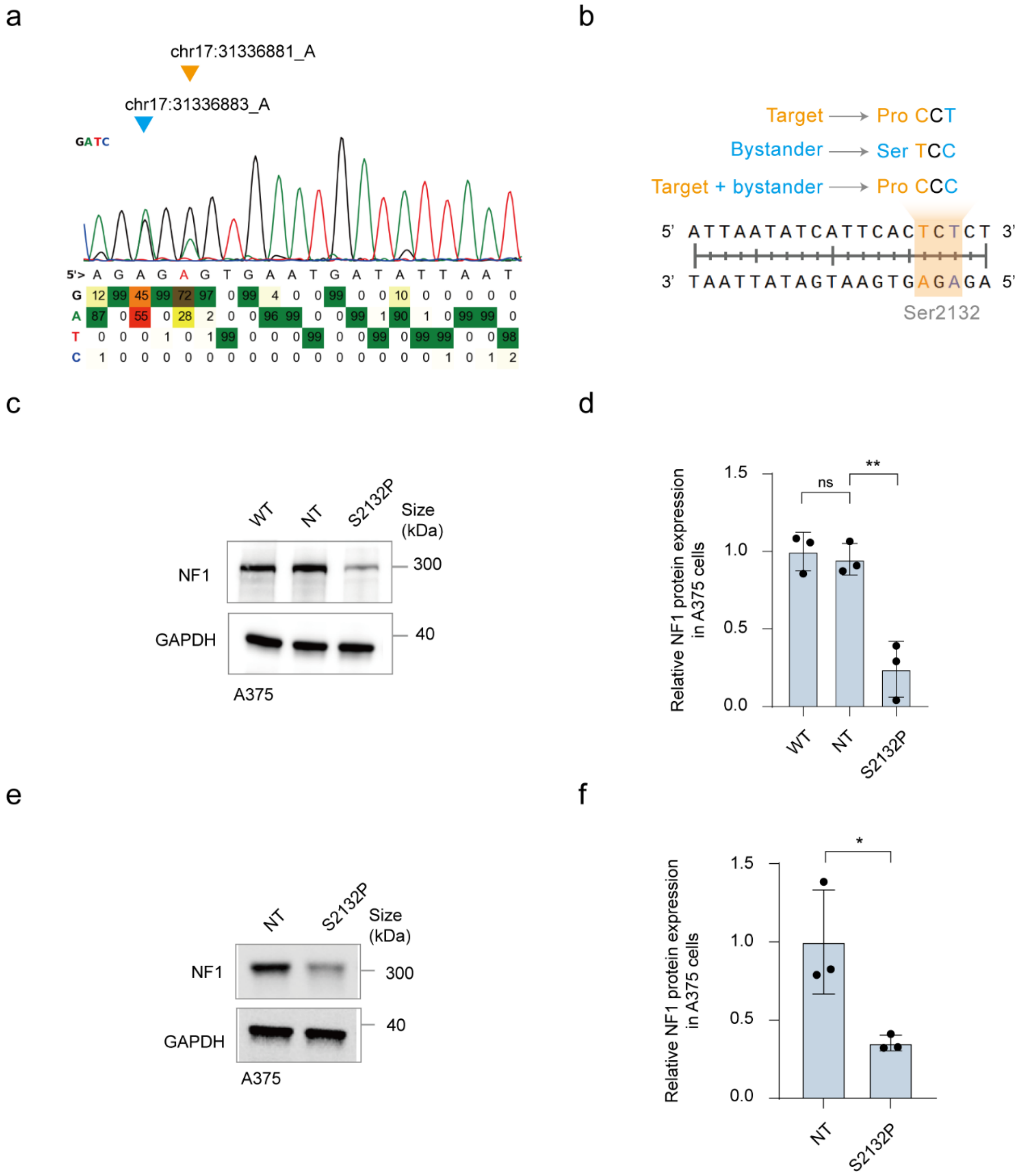

Figure S3. Orthogonal validation of reduced neurofibromin abundance in adenine base-edited A375 cell pools. (**a**) Representative Sanger sequencing chromatogram and BEAT analysis of the endogenous *NF1* target region in the adenine base-edited A375 cell pool. The target edit at chr17:31,336,881 and the

bystander edit at chr17:31,336,883 are indicated by orange and blue arrowheads, respectively. (**b**) Schematic representation of the potential editing outcomes within the Ser2132 codon. The target edit alone generates CCT, encoding proline; the bystander edit alone retains TCC, encoding serine; and the combined target and bystander edits generate CCC, also encoding proline. (**c**) Representative Western blot showing neurofibromin abundance in wild-type (WT), non-targeting control (NT), and p.Ser2132Pro (S2132P) A375 cell pools generated by adenine base editing. GAPDH was used as a loading control. (**d**) Densitometric quantification of neurofibromin abundance in (**c**), normalized to GAPDH and expressed relative to WT cells. (**e**) Representative Western blot of NT and S2132P A375 cell pools using a second NF1 antibody recognizing an independent epitope. GAPDH was used as a loading control. (**f**) Densitometric quantification of neurofibromin abundance in (**e**), normalized to GAPDH and expressed relative to NT cells. Raw densitometry values are provided in Table S7. Data are presented as mean ± SD from three independent experiments, with individual values shown. ns, not significant; * $p < 0.05$; ** $p < 0.01$, two-tailed Student's t-test.

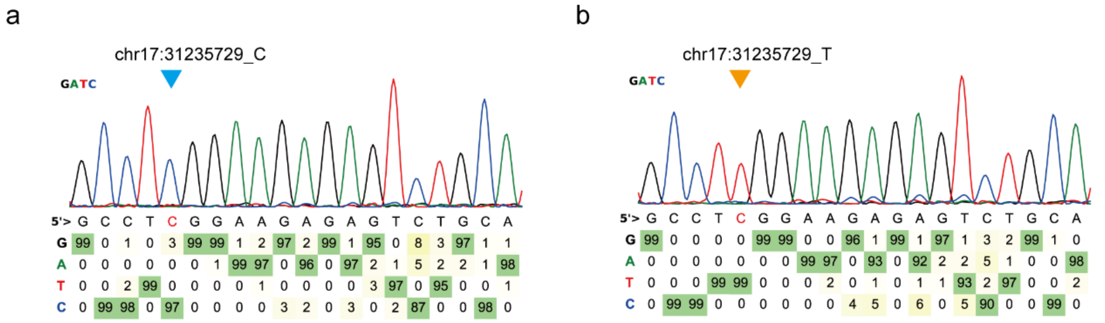

Figure S4. Sanger sequencing validation of the prime-edited *NF1* c.3827G>A (p.Arg1276Gln) variant at the endogenous locus in HEK293T cells. Representative Sanger chromatograms spanning chr17:31,235,729 (GRCh38) in (**a**) NT control HEK293T cells and (**b**) HEK293T cells prime-edited to carry *NF1* p.Arg1276Gln (R1276Q). Sequencing was performed on the antisense strand, so the targeted nucleotide reads as C in NT controls (blue arrowhead, **a**) and as T in edited cells (orange arrowhead, **b**), corresponding to the c.3827G>A substitution on the sense strand.

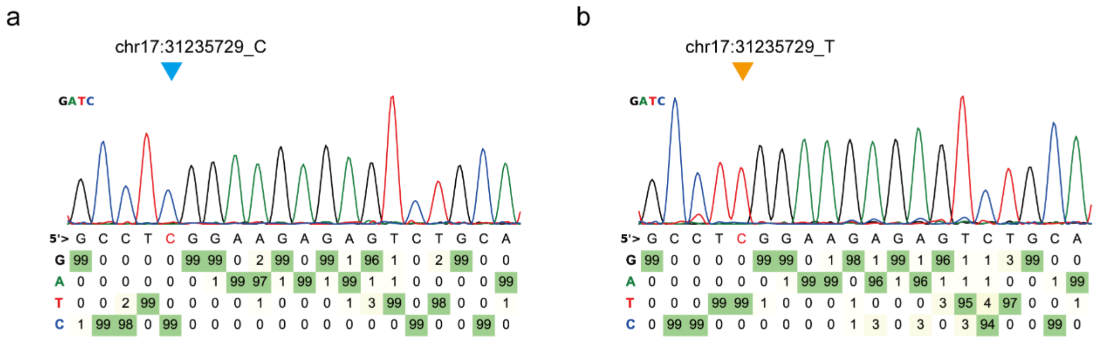

Figure S5. Sanger sequencing validation of the prime-edited *NF1* c.3827G>A (p.Arg1276Gln) variant at the endogenous locus in A375 cells. Representative Sanger chromatograms spanning chr17:31,235,729 (GRCh38) in (**a**) NT control A375 cells and (**b**) A375 cells prime-edited to carry *NF1* p.Arg1276Gln (R1276Q). Sequencing was performed on the antisense strand, so the targeted nucleotide reads as C in NT controls (blue arrowhead, **a**) and as T in edited cells (orange arrowhead, **b**), corresponding to the c.3827G>A substitution on the sense strand.

Figure S6. Uncropped western blot images corresponding to Figures 2, 3, 4 and S3.

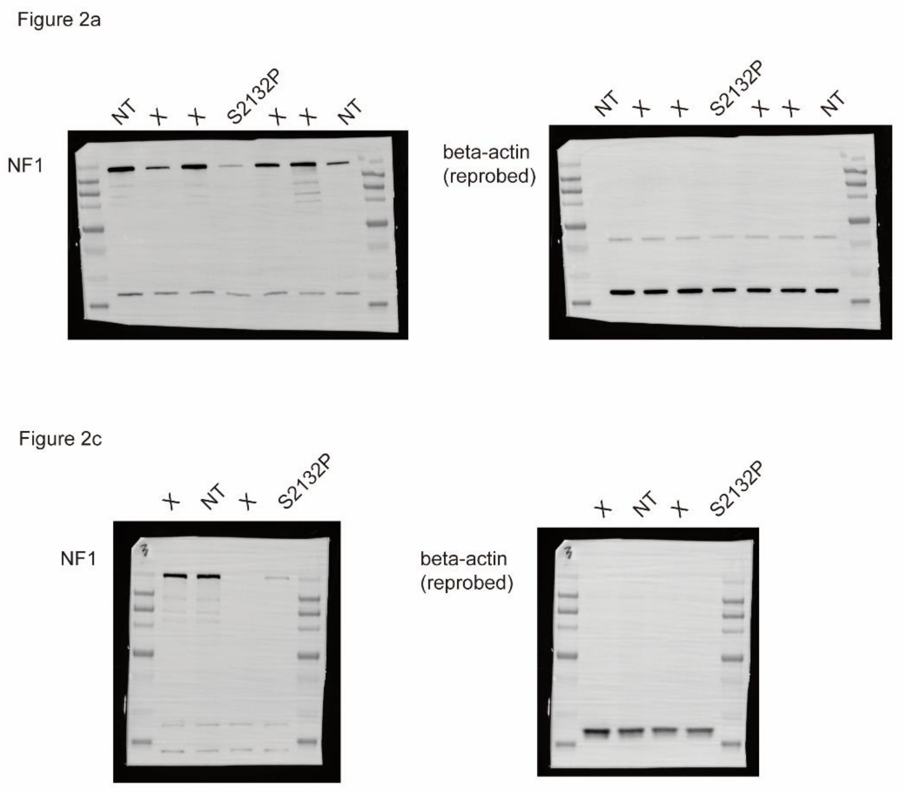

Figure 3a

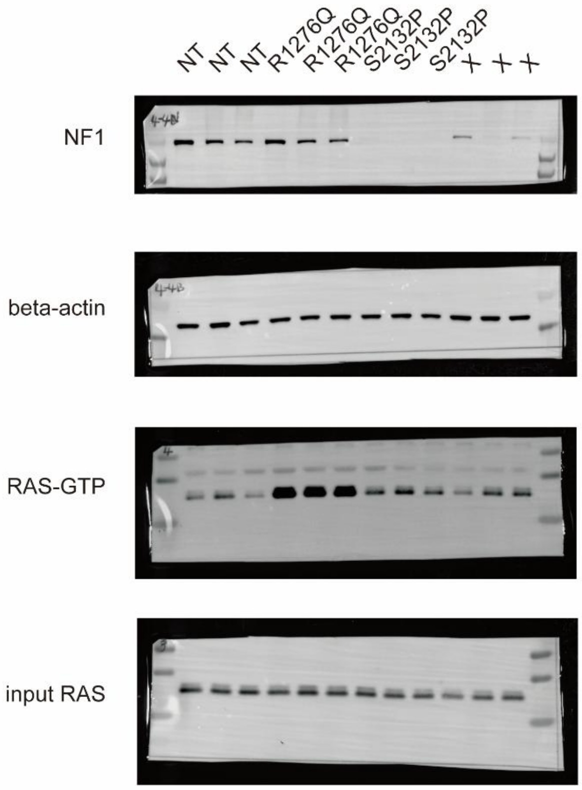
NT
NT
NT
R1276Q
R1276Q
R1276Q
S2132P
S2132P
S2132P
X
X
X
NF1
beta-actin
RAS-GTP
input RAS

Figure 4a

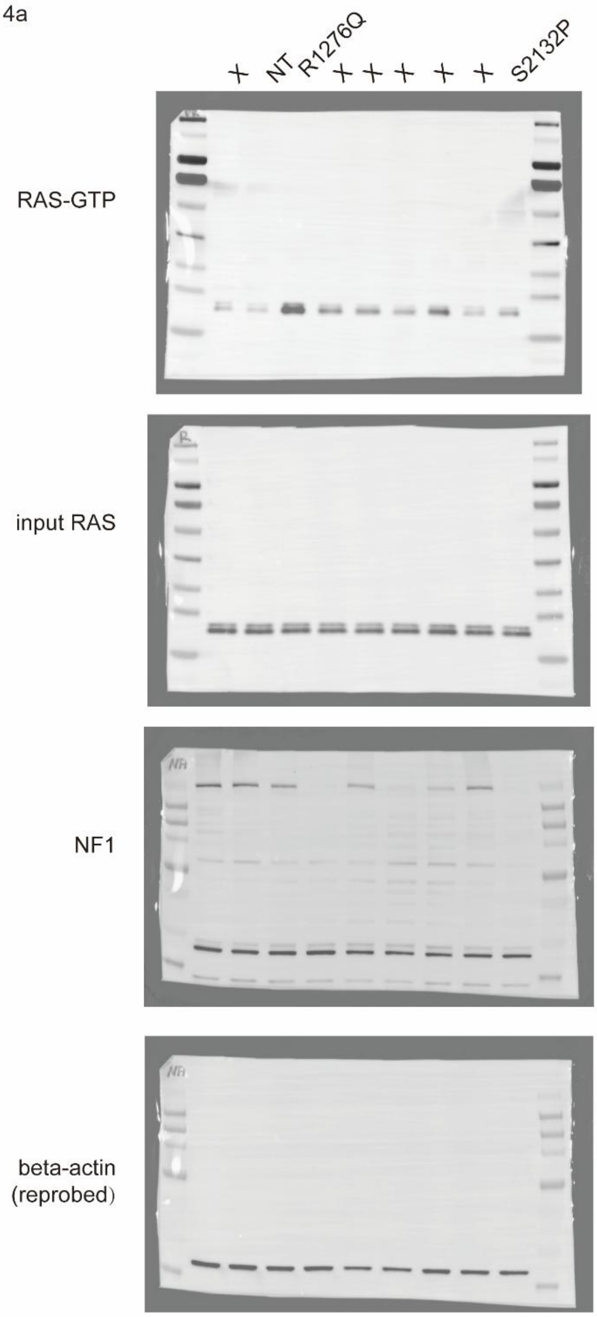
X
NT
R1276Q
X
X
X
X
X
S2132P
RAS-GTP
input RAS
NF1
beta-actin
(reprobed)

Figure 4c

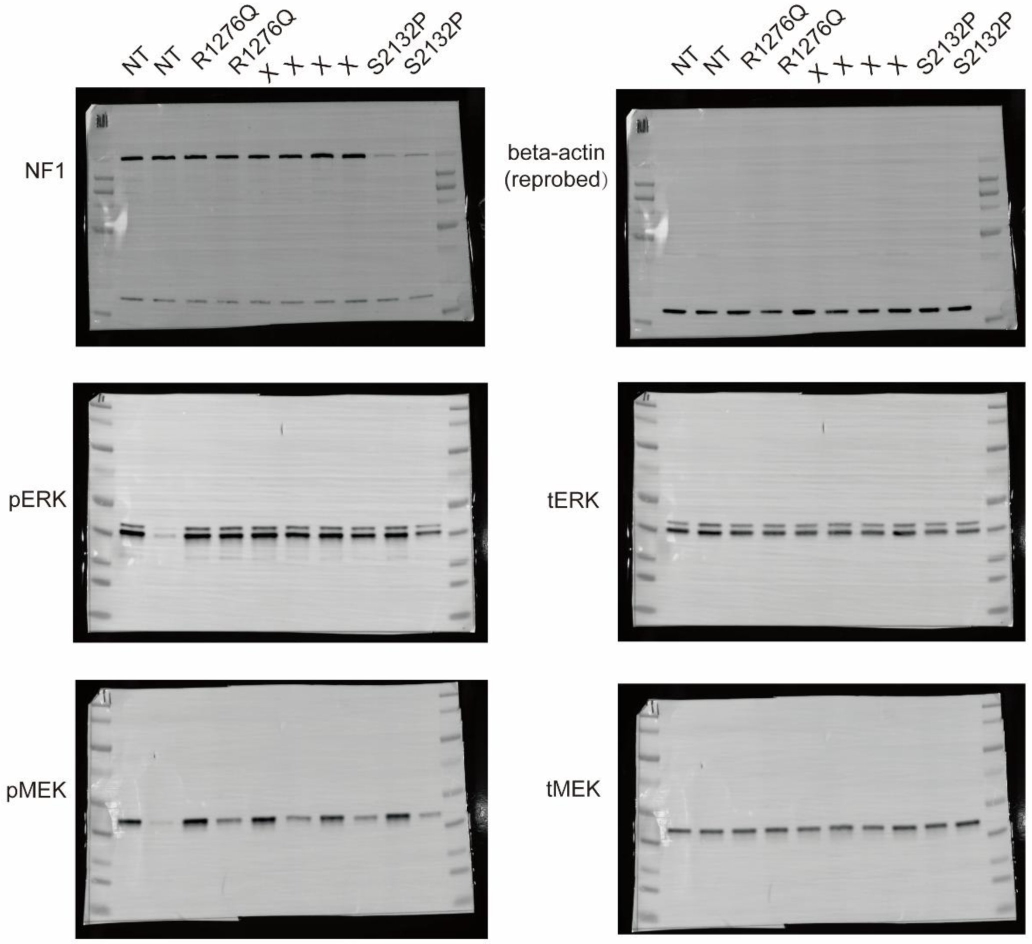
NT
NT
R1276Q
R1276Q
X
X
X
X
S2132P
S2132P
NF1
beta-actin
(reprobed)
pERK
tERK
pMEK
tMEK

Figure S3c

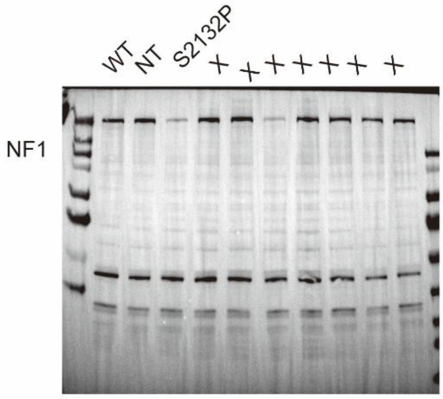

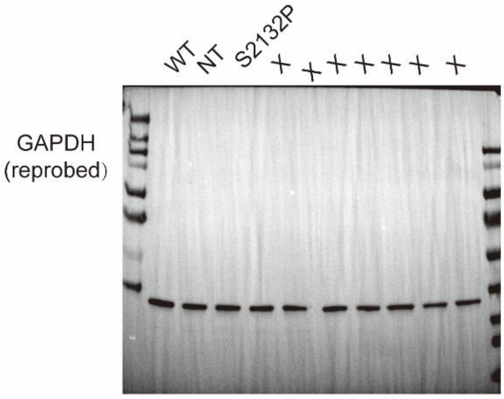

Figure S3e

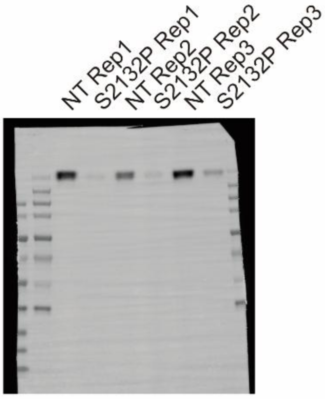

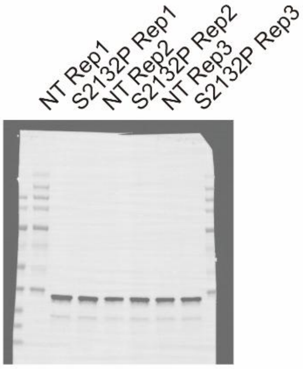

Supplement: Supplementary file 1 [file genes-17-00838-s001.zip › Supplementary Figures.pdf]
